# Removal of Sub-Internal Limiting Membrane Hemorrhage Secondary to Retinal Arterial Macroaneurysm Rupture: Internal Limiting Membrane Non-Peeling Technique

**DOI:** 10.3390/jcm12093291

**Published:** 2023-05-05

**Authors:** Akari Kimura, Hisanori Imai, Yukako Iwane, Maya Kishimoto, Yasuyuki Sotani, Hiroko Yamada, Wataru Matsumiya, Akiko Miki, Sentaro Kusuhara, Makoto Nakamura

**Affiliations:** Department of Surgery, Division of Ophthalmology, Kobe University Graduate School of Medicine, 7-5-2 Kusunoki-cho, Chuo-ku, Kobe 650-0017, Japan

**Keywords:** retinal arterial microaneurysm rupture, submacular hemorrhage, sub-internal limiting membrane hemorrhage, full-thickness macular hole

## Abstract

The appropriate surgical technique to improve the closure rate of perioperative full-thickness macular hole (FTMH) secondary to submacular hemorrhage (SMH) with sub-internal limiting membrane (ILM) hemorrhage caused by retinal arterial macroaneurysm (RAM) rupture remains an unsolved clinical problem. Several ILM transplantation techniques have been attempted, but these are challenging. Our new technique can remove sub-ILM hemorrhage with the central fovea ILM intact, without peeling the ILM. The medical records of three eyes from three patients with SMH and sub-ILM hemorrhage secondary to RAM rupture were retrospectively reviewed. During the surgery, a small ILM fissure was made outside the central fovea with ILM forceps, and sub-ILM hemorrhage was washed out through it by manually spraying balanced salt solution. Sub-ILM hemorrhage removal was achieved successfully in all eyes, with no occurrences of FTMH or other complications. Best-corrected decimal visual acuity improved from 0.05 (Snellen equivalent (SE), 20/400), 0.05 (SE, 20/400), and 0.05 (SE, 20/400) preoperatively to 0.3 (SE, 20/63), 0.4 (SE, 20/50), and 0.15 (SE, 20/125) at 3 months postoperatively, respectively. This new technique may help keep the foveal ILM intact and prevent perioperative FTMH formation.

## 1. Introduction

Submacular hemorrhage (SMH) is an ophthalmic emergency condition that results in an acute loss of vision [1]. The underlying causes include age-related macular degeneration, retinal arterial macroaneurysm (RAM), high myopia, and trauma [2].

Among the underlying causes of SMH, RAM is common in women aged ≥60 years with hypertension and atherosclerosis [3]. SMH secondary to RAM can be treated conservatively with intravitreal injection of an expandable gas and tissue plasminogen activator (tPA) or surgically with pars plana vitrectomy (PPV) combined with subretinal injection of the above drugs [4]. Previous research comparing the outcomes has revealed that SMH removal rates were not significantly different between the two treatment approaches [5]. On the other hand, the efficacy of combined treatment has also been reported [6]. Moreover, another study has recently reported favorable outcomes in that SMH removal was achieved in all cases by stratifying the layer where SMH was present, prior to determining the treatment [7]. Thus, the optimal treatment strategy for SMH associated with RAM rupture has not been clearly defined.

Bleeding associated with RAM rupture extends to various layers such as those of the subretinal space, intraretinal space, sub-internal limiting membrane (ILM), and vitreous cavity. Among them, as a treatment for sub-ILM hemorrhage, the drainage of premacular subhyaloid hemorrhage into the vitreous cavity with Nd:YAG laser photodisruption can be selected [8,9], in addition to the above-mentioned conservative treatment and surgical treatment, but surgical treatment tends to be preferred. However, sub-ILM hemorrhage itself is a risk factor for perioperative full-thickness macular hole (FTMH) formation [10], and RAM rupture-associated FTMH is considered treatment-resistant. Therefore, surgical treatment tends to be prioritized, but for the improvement of the closure rate of this refractory FTMH, careful consideration is required for handling the ILM during surgery.

Recently, several membrane transplantation techniques have been reported to improve the closure rate of FTMH by setting the ILM flap over the central fovea [9,10]. However, these techniques require very delicate manipulation of the ILM and advanced surgical skills, and in some cases, the ILM flap may be lost or misaligned, resulting in a failure to preserve the ILM over the fovea and an inability to achieve FTMH closure. Therefore, the appropriate surgical technique to reliably place the ILM flap over the fovea to improve the closure rate of FTMH secondary to RAM rupture remains an unsolved clinical problem. In addition, even if FTMH is not present during surgery, postoperative FTMH may occur in cases of RAM rupture [10]. Therefore, even to prevent the progression of postoperative FTMH formation, it may be better to finish the surgery with ILM present over the fovea. Thus, the development of better surgical techniques to reliably place the ILM flap over the fovea is urgent.

Herein, we describe a novel technique that can preserve the ILM over the fovea by removing the sub-ILM hemorrhage without peeling the foveal ILM.

## 2. Materials and Methods

This was a case series study. The medical records of three eyes of three patients with SMH secondary to RAM rupture who were treated with the new surgical technique described below were retrospectively reviewed. The study protocol was reviewed and approved by the institutional review board of Kobe University Hospital and adhered to the tenets of the World Medical Association Declaration of Helsinki. The need for written informed consent to publish this report was waived by the institutional review board. Therefore, no written informed consent procedures were necessary, but verbal informed consent was obtained from all patients for the publication of the details of their medical cases and any accompanying images, and is included in the medical records. Moreover, in accordance with the Declaration of Helsinki, this report does not contain any personal information that could lead to the identification of the patients.

### Surgical Technique

All patients were referred to our clinic for the treatment of SMH with sub-ILM hemorrhage secondary to RAM rupture. In each case, recombinant tPA and alteplase (GRTPA, Tanabe Seiyaku Co., Ltd., Osaka, Japan) intravitreal injection (40,000 IU) were administered on the first visit, and the patient was instructed to rest in the supine position for the whole day, followed by intravitreal injection of 100% sulfur hexafluoride (SF_6_) gas (0.4 cc) on the next day with instructions to rest in the prone position. Patients were reexamined 1 week after the initial visit, and if SMH removal was insufficient (cases 1 and 3) or if vitreous hemorrhage was observed (case 2), surgical treatment was performed.

The 27 G PPV with a wide-angle non-contact viewing system (Resight^®^; Carl Zeiss Meditec AG, Jena, Germany) was performed using the Constellation Vision System (Alcon Laboratories, Fort Worth, TX, USA). After the core vitrectomy, posterior vitreous detachment was created intentionally, and total vitrectomy was performed if necessary. The ILM in the sub-ILM hemorrhage area outside the central fovea was cut using a 27 G vitreous cutter (case 1) or gripped using 27 G MaxGrip forceps (Griesharber^®^; Alcon Laboratories) (cases 2 and 3), and a fissure was created. Thereafter, balanced salt solution (BSS) was sprayed over the sub-ILM hemorrhage by manually pressing the plunger of the syringe. The sub-ILM hemorrhage was sufficiently dissolved by the previously injected tPA and diffused into the vitreous cavity through the created fissure. After draining the sub-ILM hemorrhage as much as possible, the presence of FTMH was evaluated using funduscopic and intraoperative optical coherence tomography (OCT) (RESCAN 700; Carl Zeiss Meditec). In this study, FTMH was highly suspected in case 1 and absent in cases 2 and 3. Additionally, preservation of the ILM on the central fovea was confirmed by funduscopic and intraoperative OCT findings. In case 2, tPA (8000 IU) was injected into the subretinal space using a 38 G subretinal needle (MedOne, Sarasota, FL, USA) because a subfoveal subretinal hemorrhage remained. Subsequently, the vitreous cavity was filled with 20% SF_6_ gas or air, and the operation was completed. Patients were instructed to rest in the prone position on the day of the operation and were allowed to take positions other than the supine position the next day onward. Supplementary Videos S1, S2, and S3 demonstrate this novel surgical technique in cases 1, 2, and 3, respectively.

## 3. Results

**Figure 1:**
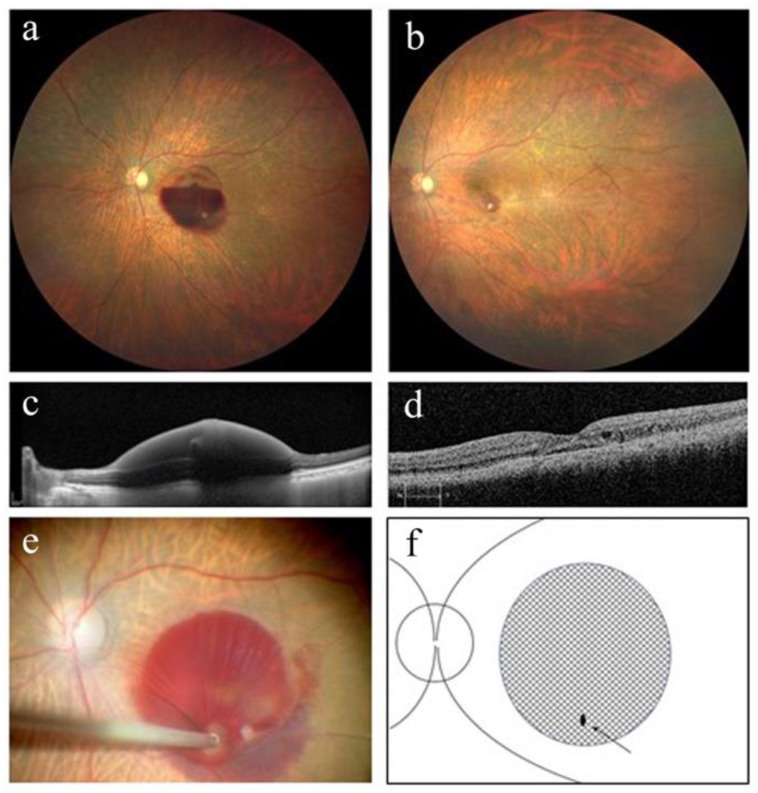
Color fundus photograph and optical coherence tomography (OCT) findings in case 1. (**a**) At the initial visit, a sub-internal limiting membrane (ILM) hemorrhage in the inferior macular region with a niveau formation was observed. (**b**) Color fundus photograph 3 months after the surgery. Retinal arterial macroaneurysm was organized, and subfoveal hemorrhage was absorbed. (**c**) OCT finding at the initial visit shows sub-ILM hemorrhage in the left eye. (**d**) OCT findings showed no full-thickness macular hole formation 3 months after surgery. (**e**) Intraoperative fundus image of the left eye. The ILM in the sub-ILM hemorrhage area outside the central fovea was cut using a 27 G vitreous cutter, and a fissure was created. (**f**) Schematic drawing of intraoperative fundus shows a fissure of ILM made by a 27 G vitreous cutter (arrow). The shaded area represents the extent of the sub-ILM hemorrhage.

**Figure 2:**
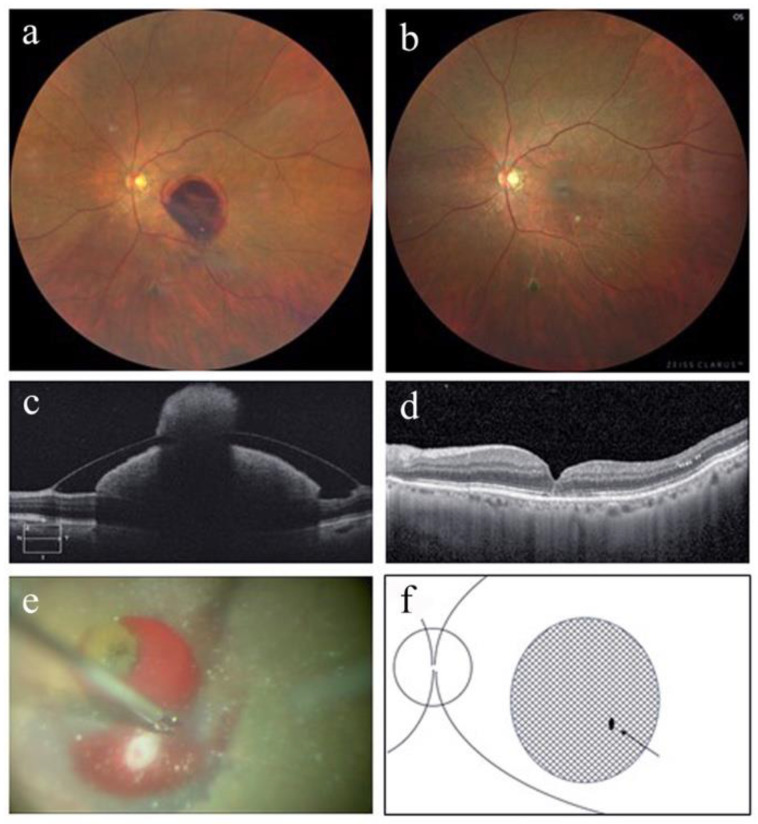
Color fundus photograph and Optical coherence tomography (OCT) findings in case 2. (**a**) At the initial visit, a subretinal hemorrhage involving the macula and a sub-internal limiting membrane hemorrhage (ILM) in the inferior macular region with a niveau formation were observed. (**b**) Color fundus photograph of the left eye 3 months after the surgery. Retinal arterial macroaneurysm was organized, and subfoveal hemorrhage was absorbed. (**c**) OCT findings at the initial visit show sub-ILM hemorrhage in the left eye. (**d**) OCT findings show no full-thickness macular hole formation 3 months after surgery. (**e**) Intraoperative fundus image of the left eye. The ILM in the sub-ILM hemorrhage area outside the central fovea was gripped using 27 G MaxGrip forceps, and a fissure was created. (**f**) Schematic drawing of intraoperative fundus shows a fissure of ILM made by 27 G MaxGrip forceps (arrow). The shaded area represents the extent of the sub-ILM hemorrhage.

**Figure 3:**
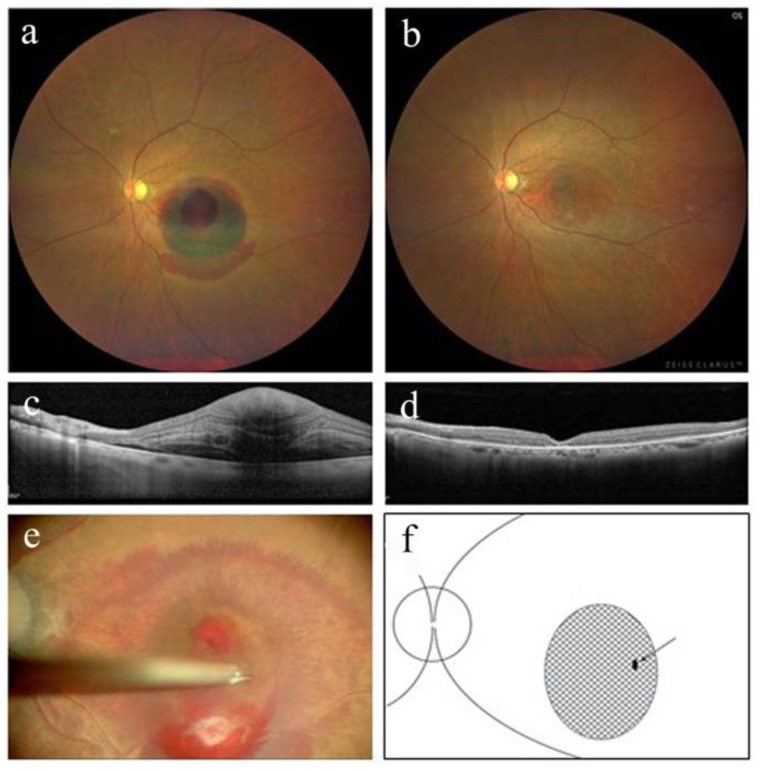
Color fundus photograph and optical coherence tomography (OCT) findings in case 3. (**a**) At the initial visit, a subretinal hemorrhage involving the macula and a sub-internal limiting membrane (ILM) hemorrhage in the inferior macular region were observed. (**b**) Color fundus photograph of the left eye 3 months after the surgery. Retinal arterial macroaneurysm was organized, and subfoveal hemorrhage was absorbed. (**c**) OCT findings at the initial visit show sub-ILM hemorrhage in the left eye. (**d**) OCT findings show no full-thickness macular hole formation 3 months after surgery. (**e**) Intraoperative fundus image of the left eye. The ILM in the sub-ILM hemorrhage area outside the central fovea was gripped using 27 G MaxGrip forceps, and a fissure was created. (**f**) Schematic drawing of intraoperative fundus shows a fissure of ILM made by 27 G MaxGrip forceps (arrow). The shaded area represents the extent of the sub-ILM hemorrhage.

Table 1 summarizes the patients’ perioperative demographic data. This study included three eyes from three women (cases 1–3) with an average age of 83.7 ± 7.2 years (92, 79, and 80, respectively). Best-corrected decimal visual acuity improved from 0.05 (Snellen equivalent (SE), 20/400), 0.05 (SE, 20/400), and 0.05 (SE, 20/400) to 0.3 (SE, 20/63), 0.4 (SE, 20/50), and 0.15 (SE, 20/125), respectively, at 3 months post operation. Figure 1, Figure 2 and Figure 3 show perioperative fundus and OCT findings in cases 1, 2, and 3, respectively. Based on fundus and OCT findings, no FTMH occurred in all cases at 3 months post operation (Figure 1, Figure 2 and Figure 3). In case 1, the activity of RAM persisted after the surgery, and direct photocoagulation for RAM was performed 1 and 2 months post operation (Figure 1). The time from the onset of symptoms to tPA intravitreal injection ranged from 1 to 4 days, and to surgery ranged from 9 to 12 days. No apparent complications were noted in any case at 3 months post surgery.

## 4. Discussion

FTMH occurs in approximately 10% of eyes with SMH secondary to RAM rupture [10,11]. It is often found intraoperatively but can also occur after PPV for RAM rupture [12]. Although the etiology is multifactorial, it is believed to occur partly due to bleeding from ruptured RAM, which flows below the retina through the structurally fragile central fovea to the vitreous cavity [13]. The outcomes of PPV with conventional ILM peeling for FTMH secondary to SMH due to RAM rupture are reported to be poorer than those for idiopathic FTMH, with a low closure rate of approximately 70% [10,13]. Therefore, methods to improve the macular hole closure rate or to prevent the occurrence of perioperative FTMH urgently need to be identified.

In recent years, the usefulness of various membrane transplantation techniques such as the inverted ILM flap technique [14,15,16,17], autologous ILM transplantation [18,19,20,21], and the ILM repositioning technique [22,23] for treating intractable macular holes (such as large macular holes, old macular holes, macular holes in eyes with high myopia, and FTMH secondary to SMH due to RAM rupture) has been reported. In a previous study on membrane transplantation for FTMH secondary to RAM rupture, Iwakawa et al. reported that autologous ILM transplantation in a patient with FTMH secondary to RAM rupture resulted in successful FTMH closure [24]. Kawaji et al. reported three cases in which the ILM repositioning technique was useful [22]. These results indicate that various types of membrane transplantation are effective for the treatment of FTMH secondary to RAM rupture. It has been reported that the presence of glial cells, such as Müller cells, within the ILM flap, which promote macular hole closure, and the production of various growth factors by the ILM flap, which further facilitate the process of macular hole closure by glial cells, were important for the success of membrane transplantation [25,26]. Thus, it is important to position the ILM on the central fovea in all methods of membrane transplantations to improve the closure rate of FTMH.

Nonetheless, in actual clinical practice, membrane transplantation requires very delicate manipulation of ILM and advanced surgical skills. The ILM flap may be lost or misaligned, resulting in failure to preserve the ILM over the central fovea; consequently, macular hole closure is not achieved. Particularly, in cases of FTMH secondary to RAM rupture, depending on the timing of the operation, the underlying ILM hematoma may develop into a blood clot and stick to the ILM, making it difficult to separate from the ILM flap. In our technique, we created a small ILM fissure outside the central fovea after the sub-ILM hematoma was adequately lysed with tPA. This procedure has three advantages: First, because tPA is used to dissolve the clots, there is no adhesion of the clots to the ILM. Second, because the central foveal ILM is not manipulated, the ILM over the central fovea can be preserved in 100% of cases. Finally, this technique is relatively simple compared to other methods. We believe this technique may be more useful than the various membrane transplantations described above in that it can preserve the foveal ILM, facilitating the closure of intraoperatively found FTMH. In addition, preserving the foveal ILM may also prevent postoperative FTMH formation, even if the presence of FTMH is not evident during surgery.

The small number of cases and the short follow-up observation period are the limitations of this study. Further studies with a larger number of cases and long-term follow-up are required to confirm our findings.

## 5. Conclusions

We report a novel PPV technique for sub-ILM hemorrhage secondary to SMH associated with RAM rupture, which can preserve the ILM over the central fovea by removing the sub-ILM hemorrhage without peeling the central foveal ILM. This technique may be useful in preventing perioperative FTMH formation.

## Figures and Tables

**Table 1:** Patient demographic data.

Case	Age (Years)	Sex	BCVA			FTMH Formation during Follow-Up	Postoperative Additional Treatment
			Pre	1 Month	3 Months		
1	92	F	20/400	20/63	20/63	No	direct photocoagulation for RAM
2	79	F	20/400	20/100	20/50	No	No
3	80	F	20/400	20/125	20/125	No	No

BCVA: best-corrected visual acuity, FTMH: full-thickness macular hole, RAM: retinal arterial macroaneurysm.

## Data Availability

The data that support the findings of this study are available from the corresponding author, H.I., upon reasonable request.

## Supplementary Materials

The following supporting information can be downloaded at: https://www.mdpi.com/article/10.3390/jcm12093291/s1, Video S1: demonstrate this novel surgical technique in cases 1; Video S2: demonstrate this novel surgical technique in cases 2; Video S3: demonstrate this novel surgical technique in cases 3.
